# A Simple Route for Purifying Extracellular Poly(3-hydroxybutyrate)-depolymerase from *Penicillium pinophilum*

**DOI:** 10.1155/2014/159809

**Published:** 2014-09-23

**Authors:** Elpiniki Panagiotidou, Constantinos Konidaris, Apostolos Baklavaridis, Ioannis Zuburtikudis, Dimitris Achilias, Paraskevi Mitlianga

**Affiliations:** ^1^Department of Mechanical and Industrial Design Engineering, TEI of Western Macedonia, 50100 Kozani, Greece; ^2^Department of Chemistry, Aristotle University of Thessaloniki, 54124 Thessaloniki, Greece; ^3^Laboratory of Biotechnology, Department of Biological Applications and Technologies, University of Ioannina, 45100 Ioannina, Greece; ^4^Laboratory of Biochemistry, Department of Biological Applications and Technologies, University of Ioannina, 45100 Ioannina, Greece; ^5^Department of Chemical and Petroleum Engineering, United Arab Emirates University, Al Ain, UAE; ^6^Depertment of Agricultural Technology, School of Agriculture Technology, Food Technology and Nutrition, TEI of Western Macedonia, Terma Kontopoulou, 53100 Florina, Greece

## Abstract

This work proposes the purification of an active and efficient enzyme, extracellular poly(3-hydroxybutyrate) (PHB)-depolymerase, suitable for industrial applications. This is achieved by the application of an easy, fast, and cheap route, skipping the chromatography step. Chromatography with one or two columns is a common step in the purification procedure, which however renders the isolation of the enzyme a time consuming and an expensive process. A strain of the fungus *Penicillium pinophilum* (ATCC 9644) is used for the isolation of extracellular PHB-depolymerase. The molecular weight of the purified enzyme is about 35 kDa and is estimated by gel electrophoresis (SDS-PAGE, 12% polyacrylamide). The enzymatic activity of the isolated enzyme is determined to be 3.56-fold similar to that found by other researchers that have used chromatography for the isolation. The as-isolated enzyme disintegrates the poly(3-hydroxybutyrate) (PHB) films successfully, as it is demonstrated by the biodegradation test results provided here.

## 1. Introduction

Plastics use in modern life has created solid-waste problems that generate skepticism for their growing use. In order to solve these problems, many methods have been employed, such as incineration, recycling, landfill disposal, and biodegradation [1]. Biodegradation is degradation caused by biological activity, particularly by enzyme action, leading to significant changes in the material's chemical structure [2]. Biodegradation may be the most effective solution for the plastic-waste problem, because it is environmentally friendly and there are no dangerous by-products. That is why, biodegradable polymers have attracted the interest of the scientific community. Biodegradable polymers (BPs) disposed in bioactive environments degrade by the enzymatic action of microorganisms such as bacteria, fungi, and algae [2].

Polyhydroxyalkanoates (PHAs) are a class of natural biodegradable polyesters with a wide range of applications, spanning from industrial to biomedical ones. PHAs, produced by a variety of microorganisms as a carbon and energy-storage source, are hydrophobic and water insoluble and are mostly degraded to water soluble products by extracellular PHAs depolymerases [3–5]. The action of PHA-depolymerases is highly specific. Each of them degrades one or two kinds of polyhydroxyalkanoates, at the most. However, PHA-depolymerases share some common properties: (i) their stability under various conditions such as pH, temperature, and ionic strength is very high, (ii) their molecular mass is relatively small (below 100 kDa), while most of them consist of only one polypeptide, (iii) they do not bind to anion exchangers such as diethyl-amino-ethanol (DEAE) at neutral pH, but they have a pronounced affinity to hydrophobic materials, (iv) the pH for their optimal activity lies in the alkaline range (7.5–9.8) and only the depolymerases of* P. pickettii* and of* P. funiculosum* have pH optima at 5.5 or 6.0, respectively, and (v) most of them are inhibited by serine hydrolase inhibitors such as diisopropylfluorophosphate or acylsulfonyl compounds, which bind covalently to the active site of serine hydrolases [3].

Polyhydroxybutyrate (PHB) is the most widely studied PHA. It is an aliphatic polyester with R configuration and physical properties similar to those of polypropylene (PP). Because of its biodegradability and biocompatibility, PHB is a very promising candidate material for biomedical applications and environmentally benign industrial applications as a substitute for PP. Bacteria and fungi secrete PHB-depolymerase (EC 3.1.1.75), which hydrolyses the ester bonds of PHB and produces oligomers and/or monomer of 3-hydroxybutyrate. These products are then metabolized to water and carbon dioxide [6, 7]. These microorganisms are ubiquitous in environments such as soil, marine water, aerobic and anaerobic sludge, and also in the human body [6]. Bacterial PHB-depolymerases have been studied in detail unlike fungi PHB-depolymerases [6, 8]. Among the few not-studied are PHB-depolymerase of* Penicillium funiculosum* and that of* Penicillium pinophilum *[9, 10]. These enzymes are composed of a single polypeptide chain, with molecular weight of about 35 kDa and optimum pH 6.0. These published experimental values are in agreement with the information taken from BRENDA (the comprehensive enzyme information system).

**Figure 1:**
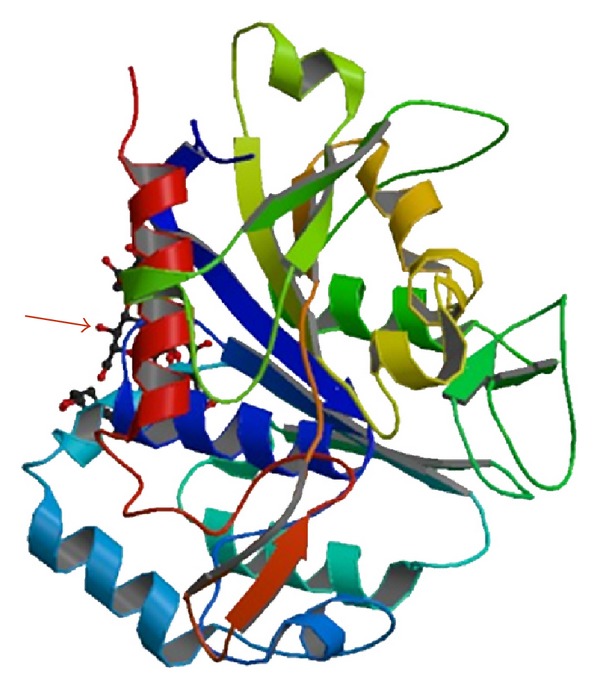
3D structure of extracellular PHB-depolymerase (National Center for Biotechnology Information (NCBI), USA).

PHB-depolymerase is composed of a catalytic domain at the N-terminus, a substrate-binding domain (SBD) at the C-terminus, and a linker region connecting these domains [8, 11]. There is a lipase-like catalytic triad (serine, aspartic acid, and histidine residues) in the catalytic domain, as well as a signature sequence of a pentapeptide [Gly-Xaa-Ser-Xaa-Gly] (where Xaa denotes any amino acid residue) known as the lipase box. Two types of catalytic domains have been identified, based on the position of the lipase box. In the type I domain, the lipase box is located in the middle of the primary structure, and, in the type II domain, it is located at the amino terminus of the structure [12]. PHB-depolymerase adsorbs onto the polymer surface using the substrate binding domain and then, using its catalytic domain, it hydrolyzes the ester bonds of the polymer chain. Accordingly, the biodegradation takes place in two steps: adsorption and scission of the ester bonds [8, 11].  Figure 1 shows the 3-D structure of extracellular PHB-depolymerase, as it is given by the National Center for Biotechnology Information (NCBI). The arrow indicates the active site.

Before attempting to understand the mechanism of an enzyme's activity, the enzyme must be purified and isolated to the point that no other enzymes can be detected [13]. Therefore, the purification and the isolation of an enzyme is a very important step, which must be designed very carefully and many factors such as pH, temperature, and metal ions presence must be considered. The purification process is considered to be successful, when the ratio of enzyme activity to the total protein is increased to the limit. For this reason, the enzyme activity and the amount of protein must be determined at every step of the procedure. The risk of failure for this process of isolation and purification, which results in isolating an inactivated enzyme, is big because enzymes are fragile and proteins can denaturalize very easily. The demand of chromatography, with one or two columns, makes the isolation and the purification of an enzyme a time consuming and expensive process and increases that risk [6, 7, 9, 10, 14–24].

In this work, the PHB-depolymerase of* Penicillium pinophilum (ATCC 9644) *was isolated and purified without using chromatography. Applying gel electrophoresis (SDS-PAGE, 12% polyacrylamide), the molecular weight of the purified enzyme was estimated to be about 35 kDa. The enzyme activity of the isolated enzyme was determined as well. The end result of this research is the use of the isolated enzyme in biodegradation studies of PHB-based products. Examining the activity and the efficiency of the isolated enzyme on the polymer's degradation is the next step.

## 2. Materials and Methods

### 2.1. Materials

Poly(3-hydroxybutyrate) (PHB) was supplied by Sigma-Aldrich Chemical Co. Prior to PHB film formation in a thermomechanical hydraulic press, PHB underwent a heat treatment in a miniextruder/compounder (HAAKE MiniLab) similar to that used, when PHB nanocomposites are prepared. The extruded PHB pieces were heated in a vacuum drying oven at 175°C for 3 min and then they were pressed in the hydraulic press under 200 atm for 3 minutes at 175°C.

All the other chemicals were supplied by Sigma-Aldrich Chemical Co. and were used as they were received.

### 2.2. Microorganism and Culture Condition

A strain of* Penicillium pinophilum (ATCC 9644) *was purchased from LGC Standards GmbH and was used for the production of the enzyme, the PHB-depolymerase. The microorganism was stored in liquid Nitrogen (LN_2_) for reuse in further enzyme production. As an initial step in the production process of PHB-depolymerase, a preculture, with a volume of 300 mL, was carried out in a mineral salt medium. The mineral salt medium contained 100 mL : 2 g Malt extract, 0.5 g Yeast extract, 40 g sucrose, 0.0012 g ZnSO_4_, 0.0004 g FeSO_4_, 0.1432 g MgSO_4_, 0.07 g K_2_HPO_4_, 0.07 g KH_2_PO_4_, 0.1 g NH_4_Cl, 0.1NaNO_3_, 0.0005 g NaCl, and 0.1 g PHB (0.1 wt%). The mineral salt medium, with pH adjusted to pH = 6.0, was sterilized in an autoclave at 121°C for 20 min. After sterilization, the inoculation of the microorganism was done at ambient temperature. The culture was incubated in a rotary shaker, under aerobic conditions, at 30°C for 3 days. After 3 days, a larger cultivation, with a volume of 1500 mL, was carried out. This bigger culture was incubated, as described previously, for 5 days. Both cultivations were carried out in flasks.

The culture was centrifuged at 18000 rpm for 30 min at 4°C and the supernatant solution was collected.

### 2.3. Purification of PHB Depolymerase

All purification procedures were carried out at the temperature range between 0 and 4°C. The supernatant solution was vacuum-filtrated using Whatman nylon membrane filters with pore sizes of 0.45 *μ*m to remove the remaining microorganism. PHB-depolymerase was precipitated by addition of ammonium sulfate. For the saturation of ammonium sulfate, the following concentrations were tested: 40%, 45%, 50%, 55%, 60%, 65%, and 70 wt%. It was found that, at 55 wt% saturation, the maximum amount of enzyme was precipitated. The precipitate was collected by centrifugation at 18000 rpm for 45 min and was dissolved in appropriate volume of 10 mM acetate buffer (pH = 6.0). The protein solution was assayed for enzyme activity and protein concentration by the methods described below.

### 2.4. Enzyme Assay

PHB-depolymerase's activity was assayed by the turbidimetric method. Initially, a stable suspension of PHB was prepared. 0.7 mg PHB was dissolved in 1 mL acetate buffer (pH = 6.0) by sonication at 20 KHz for 10 min. The reaction mixture consisted of substrate (suspension of PHB) and enzyme solution in proportion 3/1 by volume. The suspension of PHB was used as a blank. Both mixtures, blank and test samples, were incubated at 40°C for 20 min. After the incubation, their absorption at 650 nm was measured using a spectrophotometer (HITACHI, U 2800**). **The enzyme activity was calculated by the decrease in PHB turbidity. One unit of PHB-depolymerase activity is defined as the degradation of 1 mg PHB per min [6].

### 2.5. Protein Determination

The Bradford assay was employed for the quantitative determination of protein. Bovine serum albumin (BSA) was used as the standard.

### 2.6. Molecular Weight Determination

The enzyme's molecular weight was determined using gel electrophoresis (SDS-PAGE, 12% polyacrylamide) according to Laemmli method. After the electrophoresis, the gel was stained with Coomassie brilliant blue R-250.

### 2.7. The Biodegradation of PHB Films by the Enzyme

A PHB film and 4 mg of enzyme were incubated in a solution of buffer Tris-HCl (pH = 7.4) for 2 days in an oven at 37°C. After 2 days, the solution was replaced by a new one and the system (film and solution) was placed in the oven for 2 more days. After that, the films were rinsed with deiodized water and placed in a vacuum drying oven at 37°C for 24 h. The mass before and after the interaction of the enzyme with the PHB film was measured and the differences in weight were used for deducing the biodegradation rate of the film. In addition, the surface of the PHB films before and after their exposure to the enzyme solution was investigated with the aid of scanning electron microscopy (SEM). All images were recorded using a JEOL 6610 LV SEM. Prior to the SEM observations, all samples were coated with gold (Au) using Au sputtering device (Quorum 150R S) in order to eliminate charging under the electron beam.

## 3. Results and Discussion

The enzyme production depends on the culture time and the temperature at which the microorganism grows. According to the literature, the maximum quantity of PHB-depolymerase is being produced in the stationary phase at 30°C [10, 24]. The microorganism was cultured at 30°C for 8 days and then the purified enzyme was examined.  Table 1 shows the purification results of extracellular PHB-depolymerase from* Penicillium pinophilum (ATCC 9644).*

The enzyme activity increased 3.56-fold after the precipitation with ammonium sulfate. Han et al. isolated extracellular PHB-depolymerase from* Penicillium pinophilum (ATCC 9644) *using three chromatography columns with purification fold 2.1 [10]. Han and Kim, who used another fungus,* Penicillium simplicissimum *LAR13, and one chromatography column, increased the enzyme activity 2.1-fold [24]. Brucato and Wong purified extracellular PHB-depolymerase from* Penicillium funiculosum* applying hydrophobic chromatography with purification fold 4.5 [9].

In this work, application of a simple purification method, just the precipitation with ammonium sulfate without chromatography, resulted in a purified enzyme with activity 3.56-fold and comparable to that obtained from the literature.

As it can be seen in Table 1, the total protein decreased tremendously. This may be due to the presence of undesirable or nonspecific protein in the crude cellular extract.

For the removal of low-molecular-weight solutes and for increasing the concentration of the protein solution, dialysis against 10 mM acetate buffer with pH = 6.0 in a cold room (~4°C) was applied.  Table 2 presents the purification results after dialysis. As it can be seen, the purification fold of the isolated enzyme has a slight increase but the yield decreases, due to the increase of the final volume after dialysis.

**Figure 2:**
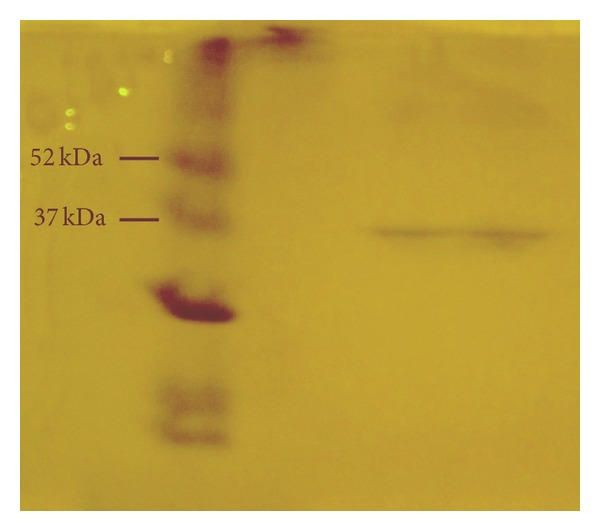
SDS-PAGE of PHB-depolymerase. The left lane shows the molecular weight markers and the right lane shows the purified enzyme.

The molecular weight of the enzyme was determined by applying SDS-PAGE electrophoresis. However, using electrophoresis one can estimate the degree of purity of a particular enzyme as well. When only a single protein band can be detected, then the purification process of the enzyme has been completed.  Figure 2 shows the results from the SDS-PAGE analysis. It can be seen that there is only one protein band. This implies that the extracellular PHB-depolymerase is pure and the process of isolation has been completed without the need of applying chromatography. The molecular weight of the enzyme was estimated to be about 35 kDa, a value in agreement with the literature [9, 10].

Since the main objective is not the isolation of the enzyme and its subsequent study for the sake of enzyme research, but it is the isolation of the enzyme and its use in biodegradation studies of PHB-based products, a purification process that is not expensive but fast and results in an active and effective isolated enzyme is highly desirable. The purification route presented here leads to the isolation of an approximately 4-fold enzyme, which effectively biodegrades the PHB films, as the biodegradation results given below prove. However, the yield of the route followed here is poor (Table 1), and this is a disadvantage of the proposed procedure. A future research with another strain of the fungus may result in higher efficiency. Concentrating the crude protein extract at first and then precipitating it with ammonium sulfate is an alternative solution to the poor efficiency problem, which will be examined in a future investigation.

**Figure 3:**
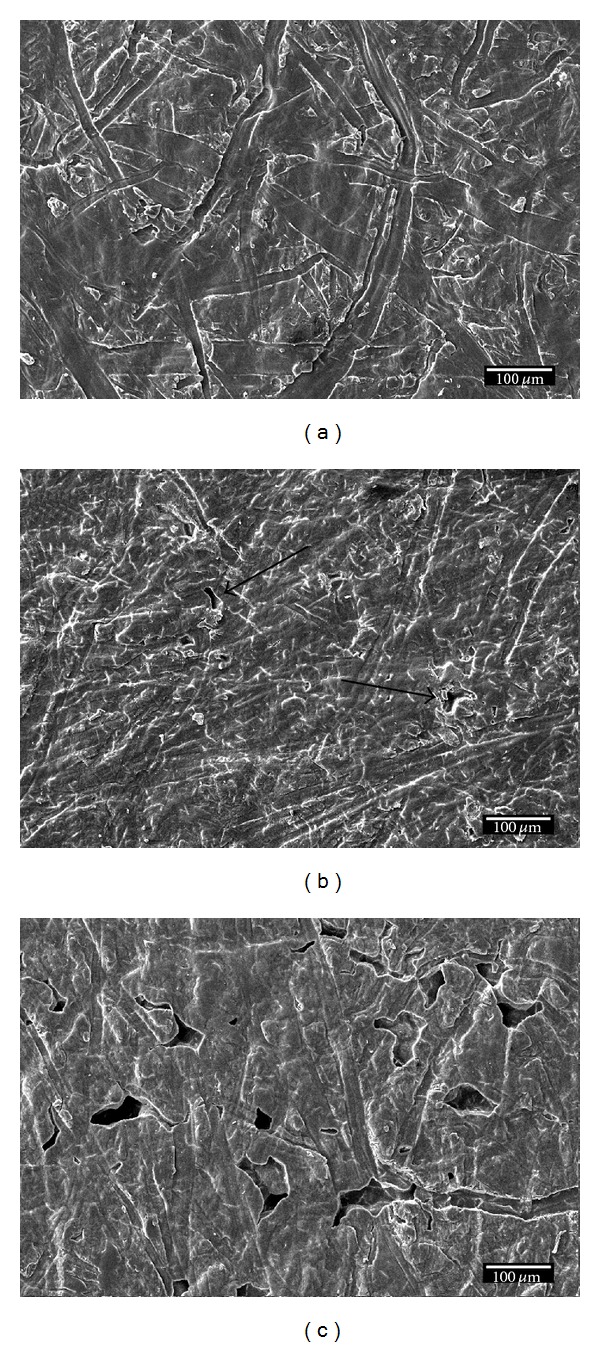
The surface of PHB before the exposure to the enzyme (a), 10 days after exposure (b), and 20 days after exposure (c).

The enzyme activity on the surface of PHB films was studied by SEM.  Figure 3 shows the surface of PHB films before the exposure to the enzyme (a), after 10 days of exposure (b), and after 20 days of exposure (c).

Before immersing the PHB film into the enzyme solution, the film is continuous, without any holes, and its surface is micron-scale rough due to the surface topography of the hydraulic press plates used for the formation of the film. Traces of erosion (indicated by arrows) appear on the surface after the immersion in the enzyme solution for 10 days (Figure 3(b)) and these traces are more evident after 20 days of exposure (Figure 3(c)). In addition, holes in the PHB film appear after its 20-day exposure (Figure 3(c)). The holes and the morphology change of the surface are due to the effect of the enzyme. The enzyme cleaves the ester bonds and disintegrates the polymer. The biodegradation rate is not too large, but this is in agreement with the literature [10]. More results on the biodegradation rates and characteristics will be presented in another work, which is under preparation.

## 4. Conclusions

An extracellular PHB-depolymerase was isolated from culture supernatant of* Penicillium pinophilum *by following a simple, quick, and inexpensive route that is attractive to industrial practices. The enzyme's activity on hot-pressed PHB films was tested in order to examine the activity and efficiency of the isolated enzyme* in situ*. The isolated enzyme has purification fold 3.56 higher than the culture supernatant. The molecular weight of the purified enzyme was determined to be 35 kDa, in agreement with the literature. The isolation of the enzyme was achieved by precipitation with ammonium sulfate and skipping the chromatography column process. This procedure is easy and fast, but its low yield is a drawback. Future work will be focused on optimizing the isolation efficiency.

## Figures and Tables

**Table 1:** Purification of PHB-depolymerase from *P. pinophilum*.

Purification step	Total protein (mg)	Total activity (Units)	Specific activity (Units/mg)	Purification (fold)	Yield (%)
Culture medium	109.095	60	0.549	1	100
Precipitation with (NH_4_)_2_SO_4_	0.7676	1.5	1.955	3.56	2.5

**Table 2:** Purification table of PHB-depolymerase from *P. pinophilum* after dialysis.

Purification step	Total protein (mg)	Total activity (units)	Specific activity (Units/mg)	Purification (fold)	Yield (%)
Dialysis	0.492	0.99	2.01	3.66	1.65

## References

[1] Franchetti SMM, Marconato JC (2006). Biodegradable polymers—a partial way for decreasing the amount of plastic waste. *Quimica Nova*.

[2] Demirbas A (2007). Biodegradable plastics from renewable resources. *Energy Sources, Part A: Recovery, Utilization and Environmental Effects*.

[3] Jendrossek D, Schirmer A, Schlegel HG (1996). Biodegradation of polyhydroxyalkanoic acids. *Applied Microbiology and Biotechnology*.

[4] Behrends A, Klingbeil B, Jendrossek D (1996). Poly(3-hydroxybutyrate) depolymerases bind to their substrate by a C-terminal located substrate binding site. *FEMS Microbiology Letters*.

[5] Ho Y-H, Gan S-N, Tan IKP (2002). Biodegradation of a medium-chain-length polyhydroxyalkanoate in tropical river water. *Applied Biochemistry and Biotechnology*.

[6] Bhatt R, Patel KC, Trivedi U (2010). Purification and properties of extracellular poly(3-hydroxybutyrate) depolymerase produced by *Aspergillus fumigatus* 202. *Journal of Polymers and the Environment*.

[7] Su-Qin C, Shan C, Liu D-B, Xia H-M (2006). An extracellular poly (3-hydroxybutyrate) depolymerase from *Penicillium* sp. DS9713a-01. *World Journal of Microbiology and Biotechnology*.

[8] Hiraishi T, Komiya N, Maeda M (2010). Y443F mutation in the substrate-binding domain of extracellular PHB depolymerase enhances its PHB adsorption and disruption abilities. *Polymer Degradation and Stability*.

[9] Brucato CL, Wong SS (1991). Extracellular poly(3-hydroxybutyrate) depolymerase from Penicillium funiculosum: general characteristics and active site studies. *Archives of Biochemistry and Biophysics*.

[10] Han J-S, Son Y-J, Chang C-S, Kim M-N (1998). Purification and properties of extracellular poly(3-hydroxybutyrate) depolymerase produced by *Penicillium pinophilum*. *Journal of Microbiology*.

[11] Fujita M, Kobori Y, Aoki Y (2005). Interaction between poly[R]-3-hydroxybutyrate depolymerase and biodegradable polyesters evaluated by atomic force microscopy. *Langmuir*.

[12] Hisano T, Kasuya K-I, Tezuka Y (2006). The crystal structure of polyhydroxybutyrate depolymerase from *Penicillium funiculosum* provides insights into the recognition and degradation of biopolyesters. *Journal of Molecular Biology*.

[13] Deutscher MP (1990). *Methods of Enzymology: Guide to Protein Purification*.

[14] Calabia BP, Tokiwa Y (2006). A novel PHB depolymerase from a thermophilic *Streptomyces* sp. *Biotechnology Letters*.

[15] Kumar A, Gross RA, Jendrossek D (2000). Poly(3-hydroxybutyrate)-depolymerase from *Pseudomonas lemoignei*: catalysis of esterifications in organic media. *The Journal of Organic Chemistry*.

[16] Liu HY, Zhang H, Chen S, Liu DB, Xia HM (2006). Purification and properties of a poly (*β*-hydroxybutyrate) depolymerase from *Penicillium* sp. *Journal of Polymers and the Environment*.

[17] Maeda H, Yamagata Y, Abe K (2005). Purification and characterization of a biodegradable plastic-degrading enzyme from *Aspergillus oryzae*. *Applied Microbiology and Biotechnology*.

[18] Nakayama K, Saito T, Fukui T, Shirakura Y, Tomita K (1985). Purification and properties of extracellular poly(3-hydroxybutyrate) depolymerases from *Pseudomonas lemoignei*. *Biochimica et Biophysica Acta (BBA)*.

[19] Papaneophytou CP, Pantazaki AA, Kyriakidis DA (2009). An extracellular polyhydroxybutyrate depolymerase in *Thermus thermophilus* HB8. *Applied Microbiology and Biotechnology*.

[20] Sang B-I, Lee W-K, Hori K, Unno H (2006). Purification and characterization of fungal poly(3-hydroxybutyrate) depolymerase from *Paecilomyces lilacinus* F4-5 and enzymatic degradation of poly(3-hydroxybutyrate-co-3-hydroxyvalerate) film. *World Journal of Microbiology and Biotechnology*.

[21] Shiraki M, Shimada T, Tatsumichi M, Saito T (1995). Purification and characterization of extracellular poly(3-hydroxybutyrate) depolymerases. *Journal of Environmental Polymer Degradation*.

[22] Wang Y, Li F, Wang ZY (2012). Purification and properties of an extracellular polyhydroxybutyrate depolymerase from Pseudomonas mendocina DSWY0601. *Chemical Research in Chinese Universities*.

[23] Zhou H, Wang Z, Chen S, Liu D, Xia H (2009). Purification and characterization of extracellular poly(*β*-hydroxybutyrate) depolymerase from *Pnicillium* sp. DS9701-D2. *Polymer-Plastics Technology and Engineering*.

[24] Han J-S, Kim M-N (2002). Purification and characterization of extracellular poly(3-hydroxybutyrate) depolymerase from *Penicillium simplicissimum* LAR13. *Journal of Microbiology*.

